# Termination of Motor Nerves in the Muscles

**Published:** 1866-10

**Authors:** 


### Termination of Motor Nerves in the Muscles.

The views of Dr. Beale relative to the mode of termination of the nerves in the muscular tissue have been pretty generally accepted in this country. Most British microscopists hold with the King's College Professor in believing that the nerves have no decided termination in the muscles, but that their ultimate fibres unite in forming a network of extreme delicacy. Abroad, however, this view has met with some opposition, and especially from MM. Kuhne and Rouget, the latter of whom has just presented a memoir to the Academy of Sciences upon the above subject. M. Rouget states that the nerve-fibre ends in a sort of terminal *plate* or disc; and in answer to Dr. Beale's denial of such a mode of termination he writes: "I shall only reply, that all other observers who have devoted themselves to this subject, MM. Krause, Kuhne, Waldeyer, Englemann, and Letzerich, and still more recently, MM. Conheim and Vulpian, have all admitted the existence of the terminal plate, and its entire independence of any nervous network." Mr. Rouget laid before the members of the Academy some photographs of microscopic preparations of tissue, which he said demonstrated the following conclusions:—(1) The terminal division of the *axis cylinder* of the motor nerve-fibre constitutes by anastomosis and fusion a terminal expansion of finely granular substance identical with that of the terminal filaments of the corpuscles of Pacini, of the ultimate nervous lamina of electric plates of fishes, etc., and in immediate contact with the contractile substance of the primitive bundle. (2) This nervous expansion is traversed in every direction by minute canals, establishing a connexion between the numerous nuclei of the *plate*, and communicating probably, on the one hand, with the space intermediate between the sarcolemma and the contractile fibrillæ, and on the other hand, with the interstice between the matrix of the nervous tube and the medullary layer—an arrangement which is doubtless related to the special action of certain poisonous substances upon the terminal extremity of the motor nerves of animal life. M. Rouget's paper will be found in the *Comtes Rendus*, June 25th.—*Lancet.*

